# Stereotactic body radiation therapy use for high risk prostate cancer in the United States

**DOI:** 10.1038/s41391-020-00300-5

**Published:** 2020-11-13

**Authors:** Sagar A. Patel, Jeffrey M. Switchenko, Amar U. Kishan, Ashesh B. Jani, Trevor J. Royce, Benjamin W. Fischer-Valuck

**Affiliations:** 1 Department of Radiation Oncology, Winship Cancer Institute of Emory University, Atlanta, GA USA; 2 Department of Biostatistics and Bioinformatics, Emory University, Atlanta, GA USA; 3 Department of Radiation Oncology, University of California, Los Angeles, Los Angeles, CA USA; 4 Department of Radiation Oncology, University of North Carolina at Chapel Hill, Chapel Hill, NC USA

**Keywords:** Prostate cancer, Outcomes research

## To the Editor:

Background: Stereotactic body radiation therapy (SBRT) is increasingly utilized for localized prostate cancer (PCa). Although its use is more widely supported for low/intermediate risk disease, the 2020 National Comprehensive Cancer Network (NCCN) guidelines now endorse its use for high risk (HR) as well. Clinicodemographic factors associated with SBRT use in HRPCa in the United States remain unknown.

Methods: Men >40 years with localized HRPCa (cT3-4 or Gleason 8–10 or PSA > 20) treated with external radiation between 2004 and 2016 were identified from the National Cancer Database. Overall, 1157 men were treated with SBRT and 48,498 with external beam radiation therapy (EBRT). Chi-square/ANOVA compared clinical/demographic characteristics, Cochran-Armitage assessed utilization trends, and multivariable logistic regression (MVA) computed odds of receiving SBRT over EBRT.

Results: SBRT use for HRPCa increased over threefold from 2004 to 2016 (0.8% in 2004 to 2.8% in 2016, *p* < 0.001), which was largely driven by increased utilization in men not receiving concomitant androgen deprivation therapy (ADT; 0.7% in 2004 to 8.3% in 2016) and those with PSA > 20 as the only HR factor (1.0% in 2004 to 4.3% in 2016). The omission of concomitant ADT, clinical stage T1-2, and Gleason score 6–7 was independently associated with increased odds of SBRT receipt. In addition, medical comorbidities and longer travel distance for treatment were associated with receiving SBRT.

Conclusion: Lower clinical stage, lower Gleason score, and omission of ADT are associated with SBRT use in HRPCa. The increase in use since 2004 has dominated by men with PSA > 20 as the only qualifying HR factor.

Ultrahypofractionation using SBRT is an advanced technique delivering large radiation doses in ≤5 treatments with cost and patient convenience advantages. As treatment volumes are smaller and exclude pelvic lymph nodes, it is predominantly utilized for low/intermediate risk PCa [1, 2]. In 2020, the NCCN added SBRT to conventional/moderate radiation regimens to the list of options for HR PCa but especially when protracted radiation courses present medical/social hardship [3]. This change occurred after the randomized HYPO-RT-PC trial [4] showed non-inferiority of ultrahypofractionation compared to conventional fractionation (~40 treatments) for tumor control and toxicity in intermediate and HR PCa after 5-year follow-up. The change also proceeded an informative meta-analysis of 39 prospective studies which showed favorable 5- and 7-year biochemical control and low toxicity [5]. However, only 8% of men had HRPCa; furthermore, those studies that included HR men did not separately report outcomes by risk group. Other data specifically supporting SBRT for HRPCa are retrospective with majority <5 years follow-up [6].

While SBRT use is increasing across all PCa risk groups in the US [1], a detailed analysis of the clinicodemographic factors associated with its use in HRPCa is lacking and is the subject of this analysis.

Men >40 years with localized, NCCN-defined HRPCa (T3-4 or Gleason 8–10 or PSA > 20) treated with external radiation between 2004 and 2016 were identified from the National Cancer Database. SBRT was defined as ≥5 Gy/fraction and ≤5 fractions, non-SBRT EBRT as ≤3 Gy/fraction and total dose ≥60 Gy. Chi-square/ANOVA tests compared clinical/demographic characteristics. Cochran–Armitage test assessed utilization trends over time. MVA computed odds of receiving SBRT. Tests were two-sided with a 0.05 level of significance. Analyses used SAS 9.4 (SAS Institute Inc). This study was waived by institutional review board.

Overall, 1157 men were treated with SBRT and 48,498 with EBRT. Supplementary Table shows clinical/demographic characteristics between groups. Higher SBRT use was associated with academic treatment center, more affluent zip code of residence, higher education level, treatment in Northeast US, longer travel distance for treatment, lower T-stage, lower Gleason score, and higher PSA.

SBRT use increased over threefold from 2004 to 2016 (*p* < 0.001), which was largely driven by increased utilization in men not receiving (ADT; 0.7% in 2004 to 8.3% in 2016, *p* < 0.001) and those with PSA > 20 as the only HR factor (1.0% in 2004 to 4.3% in 2016, *p* < 0.001); trend graphs are shown in Supplementary Figure.

On MVA, omission of concomitant ADT, clinical stage T1-2, and Gleason score 6–7 were associated with increased odds of SBRT receipt (Table 1). In addition, the presence of at least one comorbidity, longer travel distance for treatment, and non-Medicaid insurance was also associated with receiving SBRT.

**Table 1:** Multivariable logistic regression defined adjusted odds ratios for receipt of stereotactic body radiation therapy (SBRT).

Covariate	Level	Odds ratio (95% CI)	*p* value
Age at diagnosis	≥65	0.86 (0.72–1.04)	0.124
	<65	–	–
Race/ethnicity	Other	0.94 (0.64–1.37)	0.741
	Hispanic	0.95 (0.67–1.33)	0.751
	Black	1.01 (0.85–1.21)	0.889
	White	–	–
Facility type	Academic program	2.16 (1.89–2.46)	**<0.001**
	Non-academic program	–	–
Zip code median household income	<$46,000	0.50 (0.42–0.59)	**<0.001**
	≥$46,000	–	–
Zip code percent without high school degree	≥29%	1.26 (0.92–1.65)	0.131
	20–28.9%	1.01 (0.83–1.25)	0.888
	14–19.9%	0.89 (0.74–1.07)	0.234
	<14%	–	–
Insurance status	Not insured	0.60 (0.32–1.14)	0.116
	Private insurance/managed care	0.67 (0.42–1.07)	0.093
	Medicaid	0.53 (0.29–0.95)	**0.034**
	Medicare	0.78 (0.49–1.23)	0.283
	Other government	0.82 (0.48–1.41)	0.473
	Insurance status unknown	–	–
Facility location (Geographic)	Northeast	1.88 (1.51–2.33)	**<0.001**
	South	1.33 (1.07–1.65)	**0.010**
	Midwest	1.03 (0.82–1.30)	0.773
	West	–	–
Distance to treatment Facility	50+ miles	3.66 (3.00–4.46)	**<0.001**
	25–50 miles	1.94 (1.61–2.33)	**<0.001**
	0–25 miles	–	–
Charlson-Deyo comorbidity score	1+	1.23 (1.04–1.46)	**0.014**
	0	–	–
Facility location (Urban/Rural)	Metro/urban	2.51 (1.36–4.63)	**0.003**
	Rural	–	–
T-stage	T3-4	0.53 (0.43–0.66)	**<0.001**
	T1-2	–	–
Receipt of ADT	Yes	0.19 (0.16–0.21)	**<0.001**
	No	–	–
PSA	>20	0.99 (0.82–1.20)	0.926
	≤ 20	–	–
Gleason score	8–10	0.56 (0.46–0.68)	**<0.001**
	6–7	–	–

Bold values indicate statistical significance *p* < 0.05.

*ADT* androgen deprivation therapy.

Similar demographic characteristics are associated with receipt of SBRT in HRPCa as other risk groups [1, 2]. In addition, in HRPCa, longer travel distances and medical comorbidities are associated with receiving SBRT, which is concordant with NCCN guidelines encouraging its use when protracted courses create medical/social hardship [3]. Furthermore, we found men treated with SBRT compared to EBRT were more likely to have ADT omitted, lower clinical stage, and lower Gleason score. In fact, the rise in SBRT use for HRPCa has been dominated by two groups: men with PSA > 20 as the only HR factor and men not receiving concomitant ADT.

These findings are relevant. First, the subset of HRPCa with PSA > 20 as the only qualifying risk factor has been shown to have lower PCa mortality, similar to those with intermediate risk PCa [7]. Thus, men with more favorable disease characteristics more often received SBRT, and retrospective studies may overestimate the safety of SBRT. Studies demonstrating effectiveness of SBRT for HRPCa, as compared to conventionally fractionated regimens with larger target margins +/− pelvic nodal treatment, should be scrutinized regarding risk stratifying variables (e.g., representative inclusion of stage T3-4 and/or Gleason 8–10) before extrapolating to all HR cases.

Second, SBRT use was significantly associated with omission of ADT, but it remains unclear if ADT was omitted due to the utilization of SBRT, or SBRT was used due the omission of ADT for other reasons, such as patient refusal or comorbidities. Although HYPO-RT-PC [4] provides level one basis for omitting ADT with SBRT in HRPCa with similar 5-year progression-free survival as EBRT, this trial did not permit ADT in either treatment arm and was published well after the timeframe of this study. Results from the ongoing PACE-C trial (NCT01584258), which is a randomized trial comparing hypofractionated EBRT (60 Gy in 20 fractions) plus ADT versus SBRT plus ADT in intermediate and HR PCa, will be informative and potentially validating. Nonetheless, the benefit of concomitant ADT with radiation for HRPCa has been well established [8–10], and underutilization of ADT with SBRT is concerning.

This analysis has limitations. First, currently available NCDB data are only available through 2016, and utilization after 2016 could not be evaluated but would be of interest given more recent publication of HYPO-RT-PC trial and NCCN-endorsement of SBRT for HRPCa. Second, the findings are subject to the inherent biases of its retrospective nature. Third, data regarding details of radiation technique, such as use of CyberKnife or linear accelerator platforms, are unavailable and may be of interest to determine where and how patients are predominantly being treated with SBRT.

In conclusion, SBRT use for HRPCa increased threefold from 2004 to 2016 and will continue to rise given recent guideline support and emerging level one evidence. Favorable disease characteristics and, more concerningly, omission of ADT were associated with receipt of SBRT over EBRT. Randomized trials comparing SBRT with or without ADT for HRPCa are needed before considering omitting ADT.

## Supplementary information

Supplemental Table and Figure
