# Supplementary material for: Stereotactic body radiation therapy use for high risk prostate cancer in the United States
Source: Prostate Cancer Prostatic Dis. 2020 Nov 13;24(2):578–81. doi: 10.1038/s41391-020-00300-5 (PMC8116349; doi:10.1038/s41391-020-00300-5)

Supplemental Table. Patient clinical and demographic characteristics between SBRT and EBRT

			Radiation group		
	**Statistics**	**Level**	**SBRT N=1157**	**EBRT N=48598**	**Parametric P-value***
Age	Median (range)		70 (43-90)	71 (40-90)	**0.002**
Race/Ethnicity	N (Col %)	White	862 (75.48)	36079 (74.98)	0.559
	N (Col %)	Black	205 (17.95)	8392 (17.44)	
	N (Col %)	Hispanic	41 (3.59)	2126 (4.42)	
	N (Col %)	Other	34 (2.98)	1522 (3.16)	
Facility Type	N (Col %)	Non-academic program	535 (46.24)	33609 (69.16)	**<.001**
	N (Col %)	Academic program	622 (53.76)	14989 (30.84)	
Zip Code Median Household Income	N (Col %)	<$46,000	526 (47.22)	28838 (61.14)	**<.001**
	N (Col %)	>=$46,000	588 (52.78)	18329 (38.86)	
Zip Code Percent Without High School Degree	N (Col %)	>=29%	194 (17.41)	8134 (17.25)	**<.001**
	N (Col %)	20-28.9%	218 (19.57)	11124 (23.59)	
	N (Col %)	14-19.9%	226 (20.29)	11591 (24.58)	
	N (Col %)	< 14%	476 (42.73)	16311 (34.59)	
Year of diagnosis	N (Col %)	2004-06	111 (9.59)	9737 (20.04)	**<.001**
	N (Col %)	2007-09	198 (17.11)	10252 (21.1)	
	N (Col %)	2010-12	341 (29.47)	11786 (24.25)	
	N (Col %)	2013-16	507 (43.82)	16823 (34.62)	

	Statistics	Level	Radiation group SBRT N=1157	EBRT N=48598	Parametric P-value*
Insurance Status	N (Col %)	Not Insured	22 (1.9)	908 (1.87)	0.106
	N (Col %)	Private Insurance/Managed Care	321 (27.74)	12479 (25.68)	
	N (Col %)	Medicaid	33 (2.85)	1651 (3.4)	
	N (Col %)	Medicare	706 (61.02)	31066 (63.92)	
	N (Col %)	Other Government	52 (4.49)	1827 (3.76)	
	N (Col %)	Insurance Status Unknown	23 (1.99)	667 (1.37)	
Facility Location (Geographic)	N (Col %)	Northeast	383 (33.1)	11574 (23.82)	**<.001**
	N (Col %)	South	384 (33.19)	16443 (33.83)	
	N (Col %)	Midwest	224 (19.36)	12924 (26.59)	
	N (Col %)	West	166 (14.35)	7657 (15.76)	
Distance to Treatment Facility	N (Col %)	0-25 miles	794 (68.86)	39758 (82.13)	**<.001**
	N (Col %)	25-50 miles	169 (14.66)	5508 (11.38)	
	N (Col %)	50+ miles	190 (16.48)	3145 (6.5)	
Charlson-Deyo comorbidity score	N (Col %)	0	963 (83.23)	41034 (84.44)	0.265
	N (Col %)	1+	194 (16.77)	7564 (15.56)	
Facility Location (Urban/Rural)	N (Col %)	Metro/Urban	1093 (99)	46193 (97.34)	**<.001**
	N (Col %)	Rural	11 (1)	1261 (2.66)	

	Statistics	Level	Radiation group		Parametric P-value*
			SBRT N=1157	EBRT N=48598	
T stage	N (Col %)	T1-2	1025 (88.59)	38873 (79.99)	**<.001**
	N (Col %)	T3-4	132 (11.41)	9725 (20.01)	
Receipt of ADT^	N (Col %)	No	653 (56.44)	8835 (18.18)	**<.001**
	N (Col %)	Yes	504 (43.56)	39763 (81.82)	
PSA	N (Col %)	>0, <=10	391 (33.79)	20039 (41.24)	**<.001**
	N (Col %)	>10, <=20	182 (15.73)	9392 (19.33)	
	N (Col %)	>20	584 (50.48)	19163 (39.43)	
Gleason score	N (Col %)	6-7	514 (44.43)	13176 (27.11)	**<.001**
	N (Col %)	8-10	643 (55.57)	35422 (72.89)	

* The parametric p-value is calculated by ANOVA for numerical covariates and chi-square test for categorical covariates.

^ ADT: Androgen deprivation therapy

Supplemental Figure. National crude rate of SBRT use shown as a percentage among patients who received external radiation (SBRT or EBRT) as their initial definitive therapy for localized high risk prostate cancer in (a) entire cohort, (b) men with ADT omitted, and (c) men with PSA > 20 as the only qualifying high risk feature.

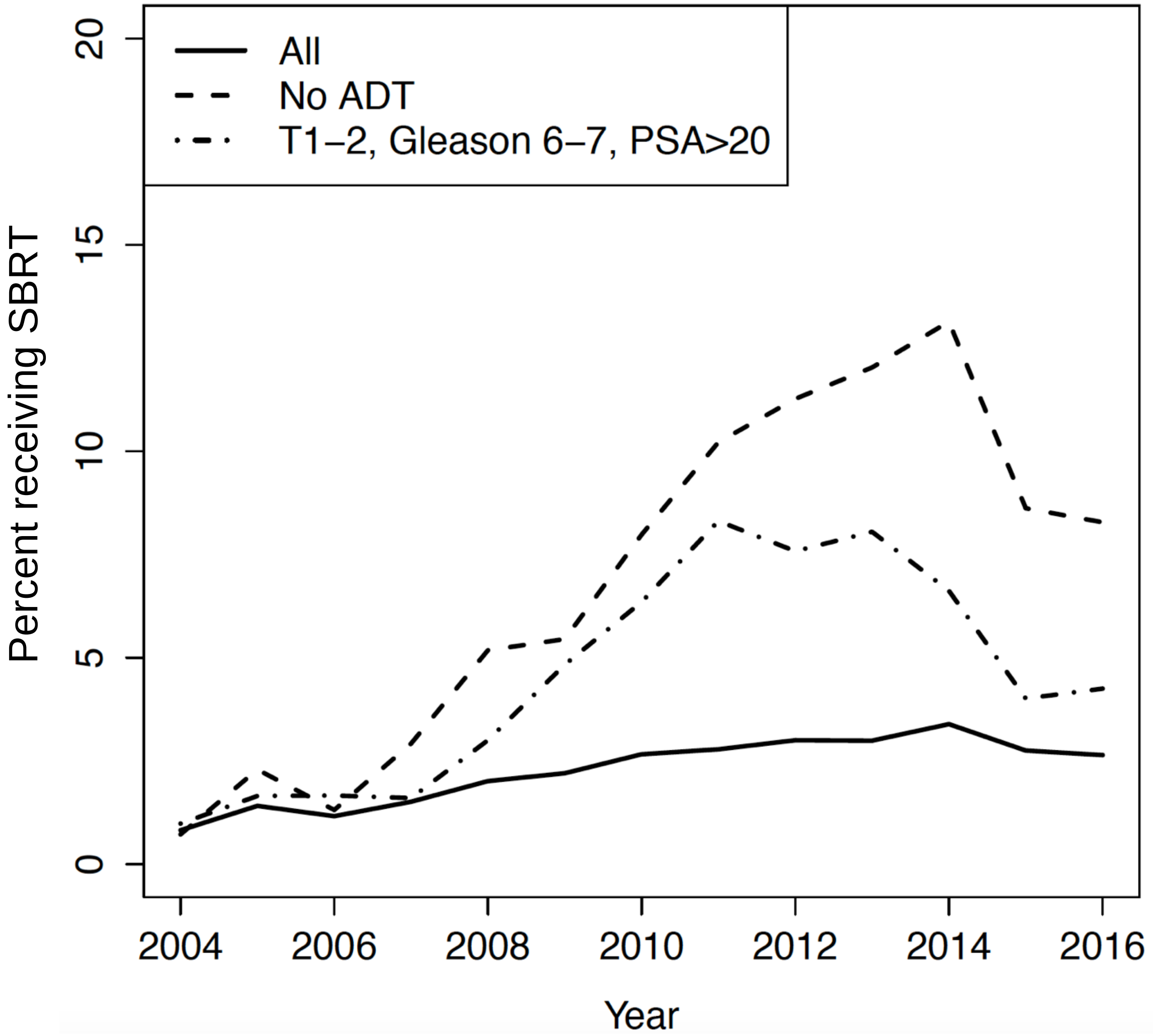

Supplement: Supplementary file 1 — Supplemental Table and Figure [file 41391_2020_300_MOESM1_ESM.pdf]
